# Supplementary material for: Anti-Helicobacter pylori Activity of a Lactobacillus sp. PW-7 Exopolysaccharide
Source: Foods. 2021 Oct 14;10(10):2453. doi: 10.3390/foods10102453 (PMC8535340; doi:10.3390/foods10102453)
Supplement: Supplementary file 1 [file foods-10-02453-s001.zip › foods-1380161-supplementary.pdf]

Anti-*Helicobacter pylori* Activity of a *Lactobacillus* sp. PW-7 Exopolysaccharide

Jingfei Hu [1,2], Xueqing Tian [1,2], Tong Wei [1,2], Hangjie Wu [1,2], Jing Lu [1,2], Mingsheng Lyu [1,2] and Shujun Wang [1,2,*]

[1] Jiangsu Key Laboratory of Marine Bioresources and Environment, Jiangsu Key Laboratory of Marine Biotechnology, Jiangsu Ocean University, Lianyungang 222005, China; jfhu123@jou.edu.cn (J.H.); xqt@jou.edu.cn (X.T.); twei@jou.edu.cn (T.W.); wuhangjie@jou.edu.cn (H.W.); jinglu@jou.edu.cn (J.L.); mslyu@jou.edu.cn (M.L.)
[2] Co-Innovation Center of Jiangsu Marine Bio-Industry Technology, Jiangsu Ocean University, Lianyungang 222005, China
* Correspondence: sjwang@jou.edu.cn

Table S1. Experiment on Removal of Hydrogen Peroxide from the Fermentation Supernatant of Strain PW-7

Indicator bacteria	Treatment methods	Diameter of bacteriostatic zone (mm)
Helicobacter pylori	Unprocessed	14.530±0.154
	Catalase treatment	14.463±0.395

Table S2. Experiment on Removal of organic acid from the Fermentation Supernatant of Strain PW-7

Indicator bacteria	Treatment methods	Diameter of bacteriostatic zone (mm)
Helicobacter pylori	Unprocessed	14.653±0.969
	pH 7.0	13.14±0.355

Table S3. Antibacterial spectrum of bacteriocin of strain PW-7

Indicator bacteria	Diameter of bacteriocin inhibition zone (mm)
Helicobacter pylori	7.843±0.131
Aeromonas salmonicida	8.153±0.263
Aeromonas hydrophila	8.060±0.337
Vibrio parahaemolyticus	7.253±0.188
Pseudomonas aeruginosa	7.207±0.031
Escherichia coli	7.127±0.261
Staphylococcus aureus	7.077±0.142

Bacillus subtilis	6.550±0.348
